# In vitro effect of two resin based materials for treating initial caries lesion around braces, under cariogenic challenge

**DOI:** 10.4317/jced.60630

**Published:** 2023-12-01

**Authors:** Maria-Gisette-Arias Provenzano, Gabriela-Cristina Santin, Daniela Rios, Sandra-Kiss Moura, Regina-Guenka Palma-Dibb, Tuane-Mertz Lucietto, Jay Bowman, Adilson-Luiz Ramos

**Affiliations:** 1 DDS, MS, PhD, Assistant Professor. Department of Dentistry, State University of Maringa - UEM, Maringá, Paraná, Brazil; 2 DDS, MS, PhD, Associate Professor. Department of Pediatric Dentistry, Orthodontics and Public Health, Bauru School of Dentistry, University of São Paulo, Bauru, Brazil; 3 DDS, MS, PhD, Assistant Professor. Department of Biophotonics Applied to Health Sciences, University Nove de Julho (UNINOVE), São Paulo, Brazil; 4 DDS, MS, PhD, Full Professor. Department of Restorative Dentistry, School of Dentistry, University of São Paulo, Ribeirão Preto, Brazil; 5 DDS, MS, PhD student. Department of Dentistry, State University of Maringa - UEM, Maringá, Paraná, Brazil; 6 DDS, Adjunct Associate Professor. Orthodontist – Private Practice, Department of Orthodontics, University of Michigan and Saint Louis University, Missouri, USA

## Abstract

**Background:**

Orthodontic treatment makes the patient susceptible to the development of white spot lesions (WSL) due to the greater accumulation of bacterial plaque. The purpose of this *in-vitro* study was to evaluate the preventive effect of two resin materials on the incidence of these lesions in the region adjacent to the fixed bracket bonded under a WSL-treated area by two resin materials.

**Material and Methods:**

36 extracted human molars presenting natural WSL were included. They were randomly divided into 2 groups for intervention: IC (Infiltration resin Icon®) and PS (resin sealant Pro Seal ®). Braces were bonded just above the WSL. The enamel surface around the brace was treated by one of the tested materials, leaving another part to be control. The samples were submitted again to a cariogenic challenge. The exposed enamel non-treated area from booth groups comprised the control group (E). For morphological analysis, confocal images were taken at baseline, after treatment, and after pH cycling of each sample. Lesion depth were measured (µm) and compared to the enamel thickness to calculate a demineralization index. All groups were compared using the Kruskal Wallis test and Dunn’s post-test.

**Results:**

PS group showed the lowest demineralization index (0.27 µm) when compared to the control area (0.39 µm) (*p*<0.05). IC presented a slightly higher percentage of lesions when compared to PS, however, did not differ significantly from the control exposed area.

**Conclusions:**

The resin sealant Pro Seal® had a positive protective effect under white spot environmental area and Icon® infiltrant, although showed some protection, did not statistically differ from controls.

** Key words:**Dental Caries, Orthodontics, Dental Enamel, Sealants.

## Introduction

Orthodontic treatment increases the risk for enamel demineralization (1,2). Bracket bonded to enamel becomes additional sites for plaque accumulation, which increases *Streptococcus mutans* and *Lactobacillus* in the presence of sucrose (2,3). As a result, initial caries lesions may occur and its prevalence may achieve up to 75% in orthodontic patients (1,3).

Fluoride-containing materials have been used as auxiliary measures to prevent initial caries lesions (4,5). Some of the preventive techniques available include fluoride varnish, acidulated phosphate fluoride gel, casein-phosphate amorphous also continued oral hygiene instructions and proper diet. Such procedures have a partial effectiveness around 40 - 50% of initial caries lesions reduction around braces (4-7).

Some authors have been suggesting treatment of those lesions, when preventing measures fail (8,9). Besides the enamel integrity, esthetic reasons justified this intention to treat (9). Infiltrating photoactivated resin materials are being used for such treatment. This technique consists in saturating the sub-surface enamel lesion by using a low viscosity resin (10-11). The Icon® resin infiltration (DMG Hamburg, Germany), uses an acid etchant -15% hydrochloric- what removes the surface layer of the subsurface initial caries lesion. This opens access to the body of the lesion which allows the resin to occlude the pores (12). Filling and occluding these pores with resin could hamper lesion progression and, more- over, should prevent cavitation by strengthening the enamel (11,12).

Desired properties of orthodontic sealants include wear abrasion resistance, adequate hardness, color stability, adequate F release, prevention of biofilm formation and resistance to acid penetration (13). Reliance Orthodontic Products (Itasca, Ill) delevoped Pro Seal®, an enamel sealant light-cured specifically for orthodontic use. The manufacturer claims a final sealant polymerization of 100% without an oxygen inhibition layer; this should eliminate any loss of material due to non-polymerization at the surface (14).

Although there are many studies about initial caries lesion filling, up until we know, there are no studies evaluating the behavior of WSL treatment when the bracket is already bonded to the enamel surface. Thus, the purpose of this study was to compare two sealants containing low viscosity resin for treating demineralized enamel sites and its behavior after bracket placement. The null hypothesis tested was that there is no difference between the treatments.

## Material and Methods

Due to the use of extracted teeth, the protocol of this *in vitro* study was approved by the UNOPAR ethics committe (number 20641213.0.0000.0108). Furthermore, donors signed an informed consent form.

The sample calculation (calculoamostral.bauru.usp.br) resulted in 17 teeth per group to obtain a power of 80%, with a significance level of α of 5%. For greater safety, 18 teeth were considered for each group. The experimental units (n = 36) were randomly divided into 2 groups according to the applied material: IC group - Icon® infiltrant (DMG Hamburg, Germany) and PS group - Pro Seal® sealant (Reliance Orthodontic Products, Itasca, III) (Table 1).

**Table 1:**
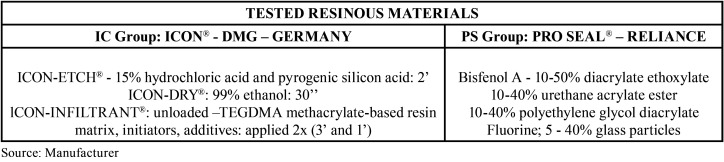
Description of the resinous materials tested.

From an initial sample of 300 extracted human third molars, 70 teeth were selected with at least one surface with a natural initial caries lesion. The exclusion criteria were the presence of spikes, hypoplasia, hypomineralization, deterioration or other superficial defects. The teeth were stored in a 0.1% thymol solution, at pH 7, at room temperature (about 22°C). Two examiners (MGAP, GS) were trained to analyze the natural initial caries lesion under ICDAS scores - 1 and 2. Only active lesions in ICDAS score 2 were included (15). Thirty-six teeth met the requirements and were reevaluated using a 4x magnifying glass (Bio-Art Equipamentos Odontológico Ltda., São Carlos-São Paulo).

**Figure 1:**
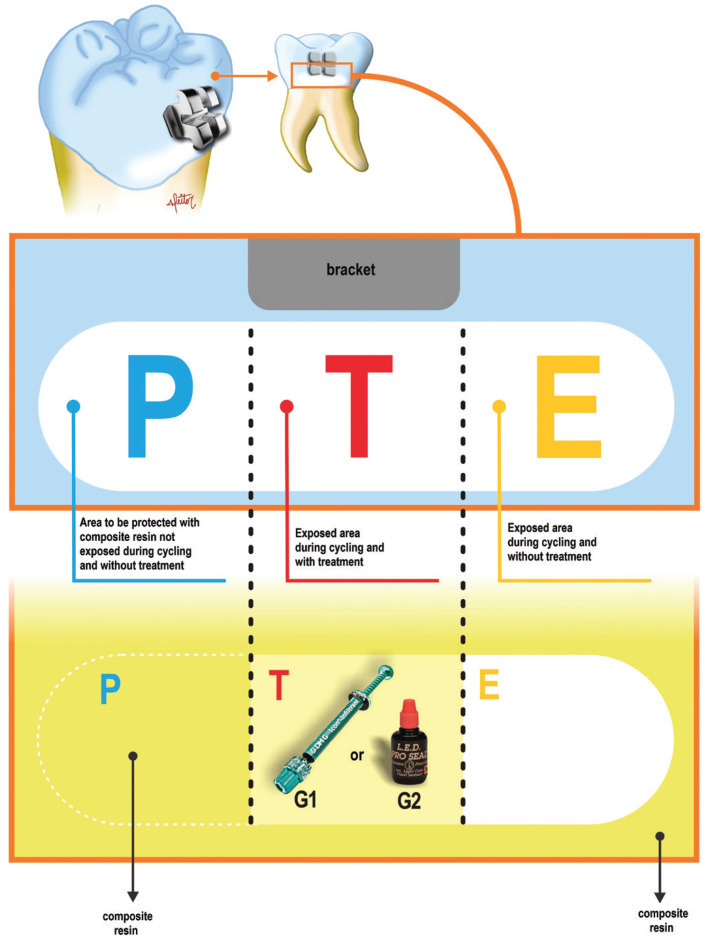
Flowchart of the reference areas of the enamel specimens for the *in vitro* test.

The enamel area under study was protected with an adhesive strip with a hole, in order to expose only the surface of interest (slightly above the location of the initial caries lesion) for bonding the orthodontic bracket (2x2mm lower incisor of the Orthometric® brand, Chicago, Illinois, USA), limiting the area ofthe acid etching under the bracket region (2 mm x 2 mm). Conditioning was carried out with 37% phosphoric acid for 15 seconds, washed for 15 seconds and dried for the same time. Then, the adhesive (Transbond-3M, Monrovia, CA, USA) was applied on the area and light cured for 20 seconds (Ultraled, Dabi Atlante, Ribeirão Preto, Brazil, 600 mw/cm2). After light curing, the adhesive strip was removed. The brackets received a layer of resin (Transbond-3M, Monrovia, CA, USA) and were pressed in place. With the use of a clinical probe, the drained excesses were removed and then the resin was polymerized for 40 seconds (20 seconds directed at the cervical interface and 20 seconds at the occlusal). The brackets were located above the natural initial caries lesion (Fig. 1).

The teeth were covered with composite resin, leaving only the area ofthe natural initial caries lesion exposed. In sequence, the image of the surface was obtained in 3D for further analysis with the laser confocal microscope. Afterwards, the second protection was performed in the first third of the natural initial caries lesion without treatment (P), necessary for the analysis of the treatment effect (area not exposed to the artificial demineralization process). Thus, the reference areas were presented in the specimen itself (Fig. 1).

To analyze the effect of the materials under a cariogenic challenge, the samples were exposed for 8 days to pH cycling (8 hours in demineralizing solution and 16 hours in remineralizing solution). The effect was evaluated on the specimen itself, comparing the protected (P), treated (T) and exposed (E) areas. The analyzes were performed in 3 stages: baseline, after treatment and cariogenic challenge.

**Figure 2:**
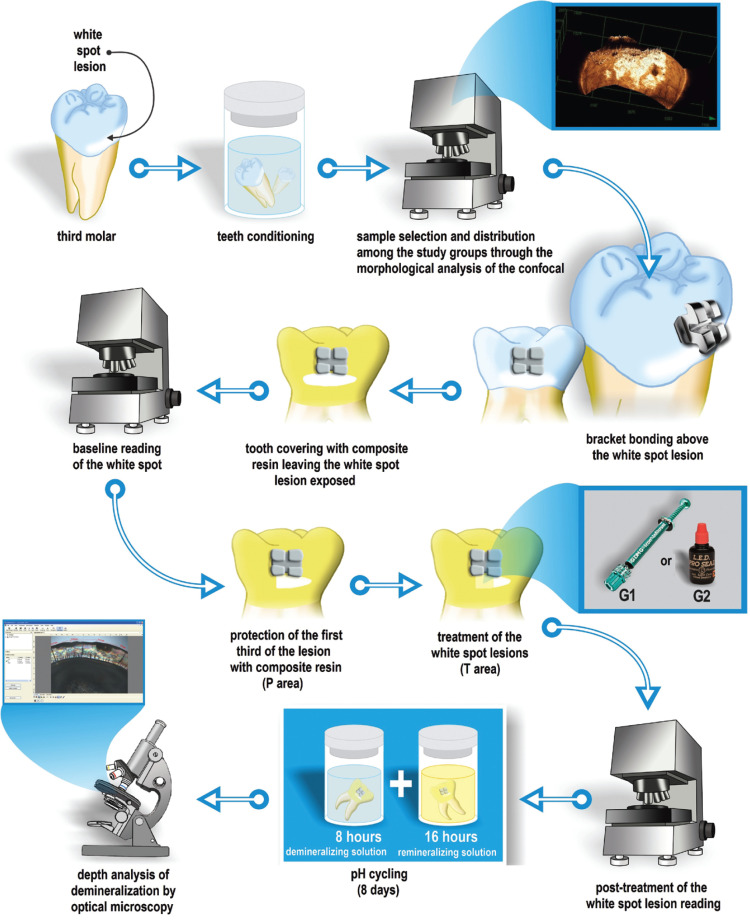
Flowchart of the experimental design.

**Figure 3:**
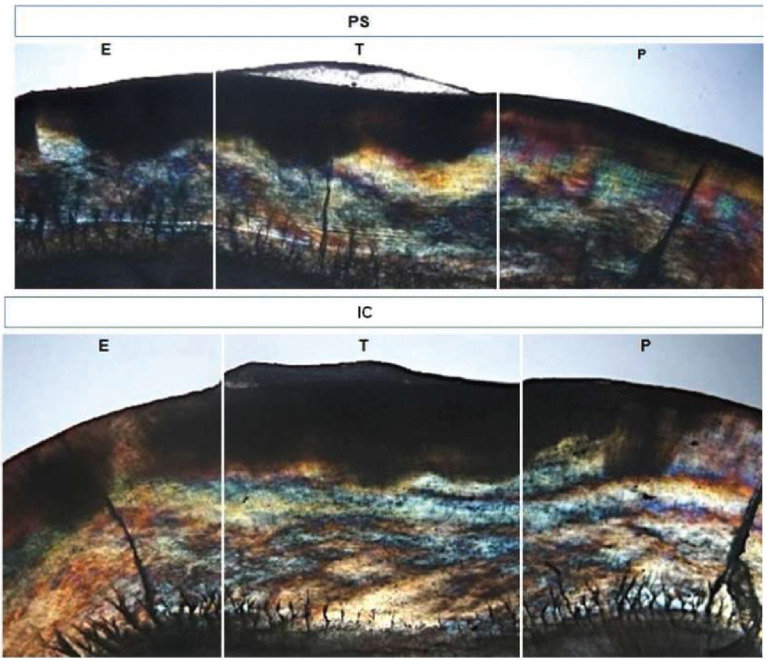
Depth of post-cycling demineralization of IC and PS groups and its extension in the exposed (E), treated (T) and protected (P) areas.

The images of the natural initial caries lesions were obtained with a laser confocal microscope (LEXT OLS 4000, OLYMPUS). To acquire the images of this study, the teeth were positioned parallel to the Table of the LEXT OLS4000® laser confocal microscope with the aid of a parallelometer. Image acquisition was performed with a 5x objective, obtaining a 107x optical zoom. Care was taken to make the posterior images always in the same region as the first image, using the references of the protected area and the orthodontic bracket. Three images of each sample were obtained, from areas P, T and E, in 3 moments during the baseline (initial) time, after treatment and after cariogenic challenge (Figs. 2,3). Image capture and profile analysis were performed after removing the resin on the enamel surface, exposing the protected area. After obtaining the initial images, the tested materials were applied below the orthodontic bracket in the central area of the lesion. Initially, a prophylaxis of the area with the lesion was performed and all residues were removed with a water jet. Then, the study region was isolated, restricted to an area of approximately 3mm2.

The IC group (n = 18) received treatment with the Icon® resinous infiltrating product (DMG, Hamburg, Germany). First, a matrix was positioned, leaving the exposed enamel window for the treatment application. Then, Icon-Etch® (15% HCl hydrochloric acid gel) was applied with a slight excess over the lesion site, for 2 minutes, followed by washing with water for 30 seconds and drying for the same time. Then, Icon-Dry® (99% ethanol) was applied on the lesion for 30 seconds, and the area was dried with an air jet for 10 seconds. The application was repeated two or three times, during 2 minutes until there was no change in the opaque whitish color of the lesion. Icon-Infiltrant® (unloaded, based on TEGDMA) with a smooth surface device was applied twice to the lesion with a slight excess, the first time acting for 3 minutes and light curing for 40 seconds (Ultraled, Dabi Atlante, Ribeirão Preto, Brazil, 600mw/cm2) and the second time, for 1 minute and again light cured for another 40 seconds. The excess material was carefully removed with a cotton roll and the surface was polished with Enhance®/PoGo silicone rubbers (Dentsply, USA) (16).

In the PS group (n = 18), Pro Seal® sealant (Reliance Orthodontic Products, Itasca, III) was applied. Initially, a matrix was positioned, leaving the window exposed in enamel for the application of the resinous agent. In the lesion area, conditioning with 37% phosphoric acid (3M) was applied for 15 seconds, washing and drying for the same period. The Pro Seal® sealant was applied with the aid of a microbrush (FGM- Joinvile, Brazil) and light cured for 40 seconds (Ultraled, Dabi Atlante, Ribeirão Preto, Brazil, 600mw/cm2).

The morphological analysis of the specimens’ surfaces consisted of depth of the demineralization. For this, images were obtained before treatment and after pH cycling with the aid of a 3D Laser Confocal Microscope (Olympus LEXT OLS 4000, Japan). Care was taken to always select part of another area of the study to have as a reference in the obtaining of the profile. The images of the time after treatment (T1t) and demineralization (T2d) were analyzed, with 5 readings that covered half the extension of the areas: *P* (reference) and T (tested) and sequentially, other 5 readings involving half of the areas: T and E. The difference between post-treatment and post-demineralization of these areas was considered.

After capturing the images using a laser confocal microscope, the teeth were sectioned using a diamond disc (#7015, KG Sorensen, Barueri, 06454-920, Brazil) adapted in a cutting machine (Minitom, Struers A/S, Copenhagen, DK-2610, Denmark), keeping the different studied areas in the same section. 0.8 mm thick sections were used for this analysis. To achieve the required thickness, the specimens were fixed in acrylic resin cylinders to plan the surface using Arotec APL-4 polishing machine (Arotec S/A Ind. e Comércio, São Paulo/SP, Brazil), with a 600 grit and 1200 grit silicon carbide sandpaper (CarbimetPaperDiscs, 30-5108-600, Buehler). Subsequently, these sections were flattened manually using sandpaper (Norton Abrasivos Ltda, São Paulo, SP, Brazil) with granulation 600 and 1200 until reaching a 150µm thickness. Then, with #600 and #1200 granulation aluminum oxide sandpaper, felt disc and 0.3 and 0.05 μm alumina suspension (Arotec S/A Ind. e Comércio, São Paulo/SP, Brazil) was used for polishing. The sections were then observed under an optical microscope (Zeiss) using deionized water as a means of imbibition. The images were captured with a 10x objective and digitalized for analysis of the enamel subsurface and with the aid of a software (AxioVision Rel. 4.8.2) it was possible to quantify the demineralized area. Ten readings were taken of the depth of the demineralization and the thickness of the enamel of the three areas (protected, treated and exposed/artificial demineralization) and then an average of each specimen/area/thickness was obtained. For data analysis, in each treated specimen the protected area was subtracted individually with the treated area (P-T) and the exposed area/artificial demineralization (P-E) and then the percentage of the demineralization lesion in relation to enamel thickness was obtained. Thus, analyzed data was the percentage of demineralization of the white spot lesion in the subsurface.

## Results

During sample preparation, 2 specimens from the IC group and 4 specimens from the PS group were discarded, because they obtained a loss greater than 0.2 mm on the enamel surface submitted to the cariogenic challenge when compared with the protected area. The percentage of the thickness of the lesion comparaded to the enamel was considered.

As data presented non-normal distribution (Shapiro-Wilk and Bartlett test), all groups were compared using Kruskal Wallis test and Dunn’s post-test, at 5% significance level.

The exposed areas of both groups were statistically compared and there was no significant difference between them (*p*>0.05). For this reason, the exposed areas both from IC and PS groups were grouped for statistic comparisons (Table 2). Although presenting slightly higher percentage of lesion depth, IC showed no significant diference when compared to PS, as well as for the control area exposed (E) to artificial demineralization. However, PS group showed lower lesion depth when compared to the exposed area (*p*<0.05) (Table 1).

**Table 2:**
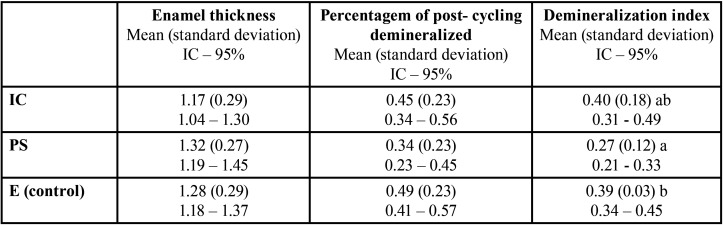
Demineralization index (percentage of post-cycling demineralized lesion (µm) and enamel thickness) comparison among the exposed area (E control) and the treated areas (groups PS and IC).

## Discussion

Aiming at minimal intervention, the use of infiltrating materials with photoactivated resin monomers has been suggested to stop the progression of incipient lesions (11,17,18). An advantage of this therapeutic scheme is the fact that it does not produce sealing margins on the tooth surface (19,20). A study observed an enamel hybridization process with the formation of resin extensions in the empty spaces in the demineralized structure when using the Icon *in vitro* (11). Furthermore, the literature demonstrates that resinous infiltration is able to preserve healthy teeth, improve the esthetics of white spot lesions, and minimize the risk of secondary caries (18). The resin infiltration can be considered an effective treatment modality for the restoration of early enamel lesions due to its good penetration property, providing better surface characteristics, and reliable masking of white spot lesions in both the dentition (21).

The progression of the lesion was analyzed according to the areas treated and exposed to the artificial demineralization process. The IC group showed no difference in the depth of the lesion between the areas. The PS group, on the other hand, exhibited a smaller lesion depth (*p*<0.05) compared to the areawithout treatment and exposed to cariogenic challenge. This result suggests that the treatment with Pro Seal sealant allowed a lower progression of the natural white spot lesion under demineralization conditions. Other studies have observed its effectiveness in preventing demineralization (14,22-25). The results also reinforce that there is no clinical evidence to support that only fully infiltrated lesions can effectively inhibit caries progression, since fissure sealants have a successful history of preventing carious lesion progression not by fully infiltrating lesions but by isolating the lesion from acidic sources (26).

Findings contrary to those observed in this study demonstrated that infiltration penetration into enamel inhibited *in vitro* demineralization (10,27,28) especially in the presence of fluoride (28). However, studies using the model of artificial caries lesion in bovine enamel has an average lesion depth of 110 µm, which is a limitation (28). In clinical situations, enamel lesions to be infiltrated into the resin are generally deeper (500- 900 µm), so specimens with natural initial caries lesions are preferable (27,28). In the present study, it was selected specimens with natural initial caries lesions with ICDAS II (distinct visual change in enamel) (15). In order to obtain a standardized sample, all selected lesions were examined with images obtained in a confocal laser microscope. Care was taken not to include specimens with natural lesions that received ICDAS II score 3 (micro cavitations in enamel) during sample selection. There is a concern with undetected microcavities on the enamel surface, as they may not be effectively filled by the infiltrant and may interfere with the success of the treatment (29).

It should be noted that the differences in demineralization patterns are extreme and do not reproduce what occurs in the oral cavity, especially in *in vitro* studies. Each technique for evaluating the tooth surface has its own limitations, and there may be destruction of the sample (which occurred when preparing some specimens for the present study). Different results can be expected when they are submitted to the complex and dynamic biological system of the oral cavity. Therefore, it is extremely necessary to carry out in situ and *in vivo* studies to expand the knowledge regarding the treatment with the Icon infiltrant and the Pro Seal sealant in non-cavitated white spot lesions.

The resin materials tested had similar behavior on the natural white spot lesion around orthodontic brackets submitted to artificial demineralization, showing similar progression of the lesions. However, Pro Seal resin sealant had a smaller demineralization compared to the control area within intra-group evaluation.
